# Life Course Association of Maternal Smoking During Pregnancy and Offspring's Height: Data From the 1993 Pelotas (Brazil) Birth Cohort

**DOI:** 10.1016/j.jadohealth.2012.08.014

**Published:** 2012-12

**Authors:** Jeovany Martínez-Mesa, Ana M.B. Menezes, David A. González, Bernardo L. Horta, Alicia Matijasevich, Denise P. Gigante, Pedro C. Hallal

**Affiliations:** a Postgraduate Program in Epidemiology, Federal University of Pelotas, Pelotas, Brazil; b Postgraduate Program in Nutrition, Federal University of Santa Catarina, Santa Catarina, Brazil

**Keywords:** Smoking, Height by age, Body height, Growth, Child, Adolescent, Cohort studies

## Abstract

**Purpose:**

To evaluate the effect of (1) maternal smoking during pregnancy; and (2) partner smoking on offspring's height in infancy, childhood, and adolescence.

**Methods:**

All hospital live births from 1993 (5,249) were identified, and these infants were followed up at several ages. Height for age, expressed as z-scores using the World Health Organization growth curves, was measured at all follow-up visits. Maternal smoking during pregnancy was collected retrospectively at birth and analyzed as number of cigarettes/day smoked categorized in four categories (never smoked, <10, 10–19, and ≥20 cigarettes/day). Partner smoking was analyzed as a dichotomous variable (No/Yes). Unadjusted and adjusted analyses were performed by use of linear regression.

**Results:**

The prevalence of self-reported maternal smoking during pregnancy was 33.5%. In the crude analysis, the number of cigarettes/day smoked by the mother during pregnancy negatively affected offspring's height in infancy, childhood, and adolescence. After adjustment for confounders and mediators, this association remained statistically significant, although the magnitude of the regression coefficients was reduced. Paternal smoking was not associated with offspring's height in the adjusted analyses.

**Conclusions:**

In addition to the well-known harmful effects of smoking, maternal smoking during pregnancy negatively affects offspring's height. Public health policies aimed at continuing to reduce the prevalence of maternal smoking during pregnancy must be encouraged.

**Implications and Contribution**

Maternal smoking during pregnancy negatively affects offspring's height in infancy, childhood, and adolescence. This association is partially explained by impaired birth weight and birth size. No effects of partner smoking on offspring's height were found.

The effect of modifiable variables early in life on several outcomes in later periods of life is an important aspect of public health. These early variables affect maternal and child undernutrition, which in turn have negative short-term and long-term consequences for the offspring's health [1–3].

According to the review published by Swanson et al. in 2009 [1], there is evidence of the harmful effects of maternal smoking during pregnancy on infant health, as well as in later stages of development. For that reason, programs to reduce maternal smoking rates have been strongly recommended [1]. Another review published in 2008 by Victora et al. [2] showed that maternal and fetal undernutrition have a negative effect on height and human capital of the offspring over the life course. In addition, maternal smoking during pregnancy has been described as associated with intrauterine growth restriction and low birth weight [4], offspring's obesity [5–9] with a dose-response effect in childhood [10], and with shorter height [11] and stunting [12].

Previous reports from the 1993 and 2004 Pelotas birth cohorts in Brazil and from the ALSPAC birth cohort in the United Kingdom found that maternal smoking during pregnancy was related to impaired linear growth in the first years of life and childhood and also was associated with height components [13] and overweight in childhood [5,6] and with psychological disorders [14]. However, Leary et al. observed that because of to the observational nature of most previous studies in this field, the possibility of residual confounding cannot be ruled out because of the social patterning of smoking [5,13].

In fact, there is a gap in the knowledge of the negative effects of maternal smoking during pregnancy, and also of partner smoking, on the height of the offspring; few studies have discussed this topic, especially using longitudinal data. The purpose of this study was to evaluate the effect of the number of cigarettes/day smoked by the mother during pregnancy and by the partner on the offspring's height. It was also aimed at evaluating the role of birth weight and birth size as mediators of this association, using longitudinal data from the 1993 Pelotas (Brazil) birth cohort study [15].

## Methods

Pelotas is a city of 340,000 inhabitants located in the extreme south of Brazil. A birth cohort study including all children whose families lived in the urban area of the city was launched in 1993. They originally comprised 5,249 individuals. Mothers were approached at the hospital, and a structured interview was carried out. Members of the 1993 Pelotas birth cohort were visited at 1, 3, and 6 months and at 1, 4, 11, and 15 years. Detailed methodologic information on the Pelotas birth cohort studies is available elsewhere [15].

Height was measured at all follow-up visits and standardized by use of World Health Organiztion growth curves, transforming it to a continuous z-score of height for age. Prenatal maternal smoking was collected retrospectively at birth by asking the mother if she had smoked during pregnancy. The number of cigarettes/day smoked by the mother during pregnancy was also collected and categorized in four categories: never smoked, <10 cigarettes/day, 10–19 cigarettes/day, and ≥20 cigarettes/day. Partner smoking was also collected retrospectively and used as dichotomous (No/Yes).

Statistical analyses were performed by analysis of variance or *t*-test for bivariate associations. After that, multivariable analyses were performed by use of linear regression. The significance level of the tests was 95% (*p* < .05). Power (1-β) was calculated after the analyses and showed values higher than 90%. Analyses using data from follow-up visits of children at 1 and 4 years old were weighed because of an oversampling of low-birth-weight children. Statistical interactions between maternal and partner smoking and also among sex were tested. Statistical models included a crude analysis, a model adjusted for confounders (model 1) and two other models adjusted for mediators: model 2 (adjusted for height-for-age z-score at birth) and model 3 (adjusted for weight-for-age z-score at birth).

Potential confounders in model 1 were as follows: income (categorized into quintiles), maternal height (centimeters), maternal age (years), and skin color (white/nonwhite). All confounders were collected at birth. All analyses including adolescents at 15 years old were adjusted for the cohort member's cigarette consumption and for pubertal status.

Informed consent was obtained from the mother or caretaker at each follow-up visit. All follow-up visits were approved by the ethical committee of the Federal University of Pelotas.

## Results

The prevalence of self-reported maternal smoking during pregnancy was 33.5% in the 1993 Pelotas birth cohort. A description of the sample is shown in Table 1 . The proportion of mothers who never smoked was 66.6% and of those who smoked ≥20 cigarettes/day during pregnancy was 5.0%. In addition, 49.5% of partners were smokers.

The mean schooling level of mothers in each maternal smoking category was as follows: never smokers, 7.2 years (standard deviation [SD] = 3.7); <10 cigarettes/day, 6.0 years (SD = 3.4); 10–19 cigarettes/day, 6.0 (SD = 3.1); and ≥20 cigarettes/day, 5.5 years (SD = 3.2) (*p* < .001). The mean maternal height was 160.0 cm (SD = 6.8), 159.3 cm (SD = 6.5), 159.8 cm (SD = 6.6), and 159.1 cm (SD = 6.8) for never smoked and <10, 10–19, and ≥20 cigarettes/day, respectively (*p* = .017) (data not shown in tables). Table 2 presents the description of the z-score height-for-age according to the number of cigarettes/day smoked by the mother during pregnancy and by the partner. A negative trend association between the number of cigarettes/day smoked by the mother during pregnancy and height-for-age z-score was detected in all ages (*p* < .001). In addition, the mean height-for-age z-score among offspring of mothers who had a smoker partner during pregnancy was lower than among mothers without a smoker partner for all ages (*p* < .001).

The results of the crude and adjusted linear regression are shown in Table 3 . In the crude analyses, the number of cigarettes/day smoked by the mother was negatively associated with height-for-age z-score in all follow-up visits (*p* < .001). After adjustment for confounders (model 1), this association remained (*p* < .001), but the value of the regression coefficients was reduced for more than 10%. After adjustment for mediators (models 2 and 3), the regression coefficients also decreased, but associations remained statistically significant (*p* < .05).

The effect of partner smoking on height-for-age z-score of the offspring was negative at all ages when crude analyses were performed (Table 3). After adjustment for confounding (model 1), the effect remained only at birth (β = −.10; −.18; −.02). This association at 1, 4, 11, and 15 years old did not remain statistically significant (*p* > .05). After adjustment for mediators (models 2 and 3), there were no effects of partner smoking on offspring's height (*p* > .05). Analyzing the statistical models (Table 3), we can observe that model 2 and model 3 explained the highest quantity of variance (all adjusted R^2^ between 17.9% and 62.9%).

## Discussion

Maternal smoking during pregnancy negatively affects the offspring's height through infancy, childhood, and adolescence, showing a negative dose-response effect of the number of cigarettes/day smoked by the mother during pregnancy on offspring's height at all ages. In addition, birth weight and birth size could be mediators in the theoretical causal chain linking maternal smoking and offspring's height. Partner smoking showed no effect on offspring's height after adjustment for confounders. The effect of the number of cigarettes/day smoked by the mother became weaker after adjustment, suggesting that confounding may play a role. The adjusted R^2^ values showed that full adjusted models explained a substantial quantity of height variance at different ages.

Previous published reports have stated that children born to smoking mothers are different from those born to nonsmoking mothers in several respects, including maternal schooling, breastfeeding, diarrhea, pneumonia, and hospitalizations [5,6,13,14]. Our results are in accordance with others previously described. An article by Matijasevich et al. using data from the Pelotas birth cohorts described the negative effect of maternal smoking during pregnancy on children's height [6]. This work evaluated prenatal maternal smoking and height in childhood, but adolescence was not evaluated, nor was the effect according to the number of cigarettes/day smoked by the mother [6]. This approach using the number of cigarettes/day smoked by the mother allowed us to evaluate a negative dose-response effect of maternal smoking during pregnancy on the offspring's height at infancy, childhood, and adolescence.

Our results show that the effect of partner smoking on offspring's height disappeared after adjustment for confounding. These findings agree with previous reports [6,13]. In other longitudinal studies that evaluated the effect of maternal smoking during pregnancy on the development of mental health problems during childhood [14], the effect of paternal smoking also disappeared after adjustment for maternal smoking. Taken together, these findings suggest that the effect of secondhand smoke is less important than the effect of maternal active smoking during pregnancy.

Leary and colleagues studied the effect of partner smoking on child body composition [5]. They described a weaker association between partner smoking and body composition of the child compared with the association with maternal smoking, and they concluded that confounding by social factors, rather than a direct effect of maternal smoking, is a possible explanation [5].

In relation to the mediating role of the newborn's anthropometrical characteristics, our results indicate that weight and length at birth are mediators in the possible chain linking the effect of maternal smoking on the offspring's height. Our results are in accordance with those of Leary et al. on the possible role of birth weight as a mediator linking maternal smoking during pregnancy and offspring's height and its components [13]. Previous reports using data from the 1993 Pelotas birth cohort indicated that after adjustment for confounding, birth weight was positively associated with height in early adolescence, pointing that an increase of one z-score in birth length was associated with a 1.63-cm increase in height at 11 years [16]. An association between birth weight and height during adolescence was reported previously by Gigante et al. in girls aged 19 years [17] but was not found in boys when they were studied at age 18 [18]. On the other hand, the negative effect of maternal smoking on intrauterine growth retardation and birth weight was demonstrated previously by Horta et al. using the 1993 Pelotas birth cohort data [4].

Biologically, children of smoking mothers have lower concentrations of mediators that stimulate fetal growth such as insulin, insulin-like growth factor (IGF) I, and IGF binding protein 3, with higher concentrations of hemoglobin and erythropoietin, perhaps attributed to fetal hypoxemia produced by maternal smoking [19]. This disadvantage apparently remains as a negative life course effect, producing impaired growth during childhood [6] and consequently reducing height and its components [13].

The prevalence of maternal smoking during pregnancy among mothers from the 1993 Pelotas birth cohort suggests a high frequency of this harmful practice. Improvements in the prenatal and overall healthcare system in Brazil in general [20,21] perhaps will improve this figure in the medium term.

Our study has some limitations. The major limitation is that the number of cigarettes/day smoked by the mother was collected by self-report. Mothers who know that smoking during pregnancy is harmful for their babies usually lie about the use of tobacco or report a lower smoking practice than their true practice [22]. Another limitation is the lack of height information about the fathers for use in the adjusted analyses.

Maternal smoking during pregnancy negatively affects the offspring's height in infancy, childhood, and adolescence and part of this effect is due to size and weight at birth. Paternal smoking has no effect on the offspring's height. Public health policies aimed at reducing the prevalence of maternal smoking must be emphasized.

## Figures and Tables

**Table 1:** Description of the participants from the 1993 Pelotas (Brazil) birth cohort study

Variables	1993	
	N	Prevalence (95% CI)
Number of cigarettes/day smoked by the mother during pregnancy		
Never smoked	3,496	66.6 (65.4;67.9)
<10 cigarettes/day	1,114	21.2 (20.1;22.3)
10–19 cigarettes/day	376	7.2 (6.5;7.9)
≥20 cigarettes/day	260	5.0 (4.4;5.5)
Partner smoking		
No	2,428	50.5 (48.5–52.5)
Yes	1,378	49.5 (46.8–52.2)
Sex		
Female	2,606	49.7 (47.7–51.6)
Male	2,642	50.3 (48.4–52.2)
Skin color		
White	4,058	77.3 (76.0–78.6)
Nonwhite	1,189	22.7 (20.4–25.3)
Family income (quintiles)		
1 (poorest)	1,031	20.1 (17.7–22.6)
2	1,195	23.3 (20.9–25.8)
3	889	17.3 (14.8–20.0)
4	1,001	19.5 (17.1–22.1)
5 (wealthiest)	1,021	19.8 (17.4–22.4)
Tanner's stage		
1	89	2.5 (.3–7.9)
2	386	11.1 (8.2–14.7)
3	1,212	34.7 (32.0–37.5)
4	1,241	35.6 (32.9–38.4)
5	561	16.1 (13.1–19.3)

CI = confidence interval.

**Table 2:** Mean of height for age z-score at different ages by number of cigarettes/day smoked by the mother during pregnancy and partner smoking: the 1993 Pelotas (Brazil) birth cohort study

Age	Variable	N	Mean (SE)	*p* value
At birth	Number of cigarettes/day smoked by the mother during pregnancy			
	Never smoked	3,406	−.38 (.02)	<.001^b^
	<10 cigarettes/day	1,089	−.78 (.04)	
	10–19 cigarettes/day	371	−.85 (.06)	
	≥20 cigarettes/day	249	−.94 (.09)	
	Partner smoking			
	No	2,379	−.42 (.03)	<.001^a^
	Yes	2,311	−.62 (.03)	
At 1 years	Number of cigarettes/day smoked by the mother during pregnancy			
	Never smoked	910	−.09 (.04)	<.001^b^
	<10 cigarettes/day	297	−.48 (.09)	
	10–19 cigarettes/day	83	−.79 (.14)	
	≥20 cigarettes/day	72	−.76 (.15)	
	Partner smoking			
	No	638	−.07 (.05)	<.001^a^
	Yes	616	−.40 (.07)	
At 4 years	Number of cigarettes/day smoked by the mother during pregnancy			
	Never smoked	833	−.05 (.04)	<.001^b^
	<10 cigarettes/day	259	−.41 (.08)	
	10–19 cigarettes/day	76	−.62 (.11)	
	≥20 cigarettes/day	63	−.75 (.12)	
	Partner smoking			
	No	577	−.07 (.05)	<.001^a^
	Yes	560	−.34 (.07)	
At 11 years	Number of cigarettes/day smoked by the mother during pregnancy			
	Never smoke	2,951	.08 (.02)	<.001^b^
	<10 cigarettes/day	951	−.11 (.03)	
	10–19 cigarettes/day	330	−.24 (.06)	
	≥20 cigarettes/day	208	−.43 (.08)	
	Partner smoking			
	No	2,064	.09 (.02)	<.001^a^
	Yes	2,023	−.10 (.02)	
At 15 years	Number of cigarettes/day smoked by the mother during pregnancy			
	Never smoked	2,738	−.06 (.02)	<.001^b^
	<10 cigarettes/day	880	−.29 (.03)	
	10–19 cigarettes/day	293	−.33 (.06)	
	≥20 cigarettes/day	183	−.46 (.07)	
	Partner smoking			
	No	1,919	−.06 (.02)	<.001^a^
	Yes	1,854	−.23 (.02)	

SE = standard error.

a *t-*test.

b Linear trend test.

**Table 3:** Height for age z-score at different ages in offspring according with number of cigarettes/day smoked by the mother during pregnancy and partner smoking: the 1993 Pelotas (Brazil) birth cohort

Model	Variable	At birth			At 1 year			At 4 years			At 11 years			At 15 years		
		N	β (95%CI)	*p* value	N	β (95%CI)	*p* value	N	β (95%CI)	*p* value	N	β (95%CI)	*p* value	N	β (95%CI)	*p* value
Crude	Number of maternal cigarettes/day		R^2^ = 2.3%			R^2^ = 3.6%			R^2^ = 3.8%			R^2^ = 1.5%			R^2^ = 1.6	
	Never smoked	5,115	0	<.001	1,361	0	<.001	1,244	0	<.001	4,440	0	<.001	4,094	0	<.001
	<10 cigarettes/day		−.39 (−.49;−.30)			−.39 (−.56;−.22)			−.36 (−.52;−.20)			−.19 (−.27;−.10)			−.23 (−.31;−.16)	
	10–19 cigarettes/day		−.47 (−.61;−.32)			−.70 (−.98;−.42)			−.57 (−.80;−.34)			−.32 (−.45;−.20)			−.27 (−.39;−.16)	
	≥20 cigarettes/day		−.56 (−.73;−.38)			−.67 (−.97;−.37)			−.70 (−.95;−.45)			−.51 (−.67;−.35)			−.40 (−.54;−.25)	
	Partner smoking		R^2^ = .5%			R^2^ = 1.6%			R^2^ = 1.2%			R^2^ = .7%			R^2^ = .7%	
	No	4,690	0	<.001	1,250	0	<.001	1,145	0	<.001	3,773	0	<.001	3,773	0	<.001
	Yes		−.19 (−.27;−.11)			−.36 (−.51;−.21)			−.26 (−.41;−.12)			−.20 (−.26;−.13)			−.16 (−.22;−.10)	
Model 1^a^	Number of maternal cigarettes/day	4,569	R^2^ = 4.6%		1,222	R^2^ = 19.1%		1,121	R^2^ = 20.0%		3,987	R^2^ = 14.5%		3,019	R^2^ = 21.5%	
	Never smoked		0	<.001		0	<.001		0	<.001		0	<.001		0	<.001
	<10 cigarettes/day		−.36 (−.46;−.26)			−.20 (−.38;−.02)			−.21 (−.38;−.03)			−.09 (−.17;−.01)			−.14 (−.21;−.06)	
	10–19 cigarettes/day		−.46 (−.61;−.30)			−.44 (−.75;−.14)			−.33 (−.58;−.09)			−.22 (−.35;−.10)			−.18 (−.31;−.06)	
	≥20 cigarettes/day		−.58 (−.77;−.40)			−.54 (−.85;−.22)			−.51 (−.78;−.24)			−.36 (−.51;−.20)			−.28 (−.43;−.13)	
	Partner smoking															
	No		0	<.001		0	.193		0	.531		0	.070		0	.399
	Yes		−.10 (−.18;−.02)			−.10 (−.25;.05)			−.04 (−.18;.09)			−.06 (−.13;.01)			−.03 (−.09;.04)	
Model 2^b^	Number of maternal cigarettes/day				1,194	R^2^ = 33.8%		1,094	R^2^ = 26.9%		3,923	R^2^ = 18.6%		2,974	R^2^ = 26.5%	
	Never smoked	–	–	–		0	.042		0	.004		0	<.001		0	.006
	<10 cigarettes/day		–			−.03 (−.20;.13)			−.11 (−.27;.06)			−.01 (−.10;.07)			−.07 (−.15;.01)	
	10–19 cigarettes/day		–			−.17 (−.46;.11)			−.18 (−.42;.06)			−.14 (−.27;−.02)			−.10 (−.22;.02)	
	≥20 cigarettes/day		–			−.29 (−.59;.01)			−.36 (−.61;−.11)			−.27 (−.42;−.12)			−.16 (−.31;−.01)	
	Partner smoking															
	No		–	–		0	.308		0	.713		0	.103		0	.815
	Yes		–			−.07 (−.21;.07)			−.02 (−.16;.11)			−.06 (−.12;.01)			−.01 (−.07;.06)	
Model 3^c^	Number of maternal cigarettes/day	4,568	R^2^ = 62.9%		1,210	R^2^ = 31.2%		1,109	R^2^ = 25.6%		3,951	R^2^ = 17.9%		2,996	R^2^ = 25.6%	
	Never smoked		0	.161		0	.044		0	.004		0	.003		0	.003
	<10 cigarettes/day		−.06 (−.13;−.01)			−.04 (−.21;.12)			−.11 (−.28;.06)			−.02 (−.10;.06)			−.07 (−.15;.01)	
	10–19 cigarettes/day		−.02 (−.12;.08)			−.17 (−.45;.12)			−.18 (−.42;.06)			−.14 (−.27;−.02)			−.10 (−.22;.02)	
	≥20 cigarettes/day		−.06 (−.17;.06)			−.30 (−.60;.01)			−.36 (−.62;−.10)			−.26 (−.41;−.11)			−.18 (−.33;−.03)	
	Partner smoking															
	No		0	.856		0	.294		0	.711		0	.130		0	.753
	Yes		−.01 (−.05;.05)			−.08 (−.22;.07)			−.03 (−.16;.11)			−.05 (−.12;.01)			−.01 (−.07;.05)	

β = linear regression coefficient; CI = confidence interval; *p* values = Wald's test for trend; R^2^ = squared correlation coefficient adjusted by degrees of freedom.

a Model 1: Adjusted for confounding (paternal smoking, family income, maternal height, maternal age, skin color). At 15 years old, analyses were also adjusted for adolescent smoking and pubertal status.

b Model 2: Model 1 + z-score height for age at birth.

c Model 3: Model 1 + z-score weight for age at birth.
